# Biochemistry of microwave controlled *Heracleum sosnowskyi* (Manden.) roots with an ecotoxicological aspect

**DOI:** 10.1038/s41598-024-65164-4

**Published:** 2024-06-20

**Authors:** Krzysztof Słowiński, Beata Grygierzec, Anna Wajs-Bonikowska, Agnieszka Baran, Sylwester Tabor, Pitr Waligórski, Magdalena Rys, Jan Bocianowski, Agnieszka Synowiec

**Affiliations:** 1 Department of Forest Utilization, Engineering and Forest Technology, The University of Agriculture in Krakow, al. 29 Listopada 46, 31-425 Kraków, Poland; 2 Department of Agroecology and Plant Production, The University of Agriculture in Krakow, al. Mickiewicza 21, 31-120 Kraków, Poland; 3 Department of Biotechnology and Food Science, Lodz University of Technology, ul. Stefanowskiego 2/22, 90-537 Lodz, Poland; 4 Department of Agricultural and Environmental Chemistry, University of Agriculture in Krakow, al. Mickiewicza 21, 31-120 Kraków, Poland; 5 Department of Production Engineering, Logistics and Applied Computer Science, The University of Agriculture in Krakow, ul. Balicka 116 B, 30-149 Kraków, Poland; 6 The Franciszek Górski Institute of Plant Physiology, Polish Academy of Sciences, Niezapominajek 21, 30-239 Kraków, Poland; 7 Department of Mathematical and Statistical Methods, Poznan University of Life Sciences, 60-637 Poznan, Poland

**Keywords:** Control, Ecotoxicology, Essential oil, Fatty acids, Roots, Sugars, Environmental chemistry, Biochemistry, Environmental sciences, Plant sciences, Plant ecology

## Abstract

Sosnowski hogweed is an invasive weed in eastern-middle Europe that is dangerous to human health and the environment. The efficacy of its control using chemical and mechanical methods is limited. Electromagnetic radiation (microwaves) could be an environmentally friendly alternative for controlling this species. This study aims to: (1) Determine the effect of varying microwave treatment (MWT) durations on the control of S. hogweed using a device emitting microwaves at 2.45 GHz, 32.8 kW/m^2^; (2) Evaluate the impact of MWT on soil by an ecotoxicological bioassays; (3) Analyze biochemical changes occurring in the roots during the process. A field study was performed to assess the efficacy of S. hogweed control using MWT in times from 2.5 to 15 min. The MWT-treated soil was collected immediately after treatment (AT) and tested using bioassays (Phytotoxkit, Ostracodtoxkit, and Microtox). Fourteen days AT, the MWT hogweed roots were dug out, air-dried, and analyzed for the content and composition of essential oil, sugars, and fatty acids. According to the ecotoxicological biotests, the MWT soils were classified as non-toxic or low-toxic. The regeneration of hogweed was observed only in non-treated plants (control). Hogweed MWT for 2.5–15 min did not regenerate up to 14 days AT. The average weight of roots in hogweed MWT for 15.0 min was ca. two times smaller than the control plants. Those roots contained significantly higher amounts of sugars and saturated fatty acids than the control. We did not find a correlation between S. hogweed root essential oil content and composition and MWT time. The main compounds of essential oil were p‑cymene and myristicin. No highly photosensitizing compounds were identified in the tested root oil. We conclude that MWT of S. hogweed could be an environmentally safe and prospective control method, but more studies are needed.

## Introduction

Sosnowski hogweed (*Heracleum sosnowskyi* Manden.) is one of the three *Heracleum* species known as giant hogweeds. This is a perennial plant from the Apiaceae family, originally from the Central and Eastern Caucasus, Transcaucasia, and Turkey^1^, but spread worldwide as an invasive plant, particularly in Europe^2,3^ and North America^4–6^. The European Union has included giant hogweeds in the Invasive Alien Species of Union Concern list^7^, requiring EU countries to reduce their spread. Invasive giant hogweeds are highly competitive with native flora. They are characterized by an intense growth rate, huge size, and a high reproduction rate^8^. In Poland, Sosnowski hogweed greatly threatens humans and the environment^9^. Its biomass contains a large amount of crude protein. Since the 1960s, it has been cultivated as a fodder in many regions of Russia: Leningrad, Moscow, Ivanovo, Kirov, Pskov, the Komi Republic, as well as in Belarus and the Baltic countries^10^. For this purpose, it was also imported to Central and Eastern European countries. However, after a short research period, its cultivation was abandoned.

Sosnowski hogweed causes health problems in humans and animals and has strong allelopathic properties for nearby plants^11^. These features are determined in S. hogweed and other *Heracleum* species due to furanocoumarins, located on the surface of hairs and in other epidermal cells of leaves and stems, as well as in parenchyma cells^12,13^, responsible for the photosensitizing, mutagenic, and carcinogenic effects. The most dangerous compounds are psoralen, bergapten, and xanthotoxin^14,15^. In the presence of UV radiation, furanocoumarins cause the so-called photodermatoses in humans, manifesting as irritation and redness caused by burns, swelling and, consequently, may lead to permanent skin discoloration and scarring^14,16^. On the other hand, these compounds have several positive, scientifically confirmed properties, such as antioxidant and anticancer^17,18^. Thus, many ingredients with aromatic, nutritional, and photosensitizing properties were detected in the seeds and roots: among others, the seeds contain significant amounts of essential oil and fat, and the roots contain isobergapten, isopimpinellin and sphondin^19^. So far, research has focused mainly on the biochemical analysis of the plant's aboveground parts: seeds, leaves, stems, and flowers^20–22^. To the best of our knowledge, there is no information on the content and composition of volatile compounds in the roots of S. hogweed, and in this regard, our work is innovative. In terms of the composition of essential oil (OE), a thematically similar publication shows the composition of essential oil from the roots of *Heracleum persicum*^23^, but it is burdened with many errors.

The popular method of S. hogweed control is the use of synthetic herbicides^24^. However, due to their negative environmental impact, they are not a recommended method, especially in natural ecosystems. In turn, mechanical methods of eliminating Sosnowski's hogweed, recommended for use in protected areas, involve cutting the plants (often multiple times during the growing season) and may only be effective in the long term^24^. Therefore, new methods of eliminating Sosnowski's hogweed are being sought.

One of the new methods of S. hogweed control could be electromagnetic radiation (microwave). That method is now common in food processing^25–27^ to improve seed germination^28^, for soil disinfection, and for drying wood and wood-based products^29,30^. Microwave radiation can stimulate the growth and development of plants^31,32^. However, as radiation frequency and plant exposure duration increase, inhibitory effects are observed^33,34^, and the main factor responsible for these effects is generated heat, which depends on exposition time to microwaves. Loner exposition of plant tissue to microwaves causes non-reversible degradation^35,36^.

For this reason, the microwave method could be classified as a thermal method of weed control. Our previous studies showed microwave radiation could potentially control Sosnowski hogweed^35^ or even perennial weed Japanese knotweed (*Reynoutria japonica* Houtt.)^36^. Compared to the other thermal methods of weed control, e.g., flame method, microwave is more environmentally friendly as it does not require gases to produce heat. Moreover, the device used in this experiment (horn antenna) is handy and light and can be applied precisely to the controlled plant. The optimal condition for using high-frequency microwave radiation is also to minimize the risks caused by spot heating in the soil environment. In the risk analysis, it is important to establish to what extent microwave radiation is a factor that harms the environment and can, therefore, be considered a stress factor for soil organisms^36^. Relatively quick verification of the environmental effect of various factors on living organisms can be carried out using ecotoxicological biotests. Biotests involve the controlled exposure of organisms to substances present in the sample and the qualitative and quantitative evaluation of these effects^37^.

In the following studies, we have expanded the spectrum of analyses to include the impact of microwaves of different time ranges on the biochemical properties of Sosnowski hogweed and the environmental effect. We also measured the content of sugars and fatty acids. These compounds were selected for detailed analysis based on the results obtained in previous studies^35^, where the metabolomic analysis of tissues and sap showed significant changes mainly in the content and composition of sugars and fatty acids. Sugars are the primary products of photosynthesis and perform multiple roles in plants, such as energy and carbon transport molecules, hormone-like signaling factors, osmoprotectants, and the source of reserve materials from which plants make proteins, polysaccharides, or oils^38^. Fatty acids are the basic components of the cell membrane. Therefore, lipids' composition and saturation/unsaturation are related to the stability of the cell membrane at high temperatures^39^. The work aimed to: (1) Determine the effect of varying MWT durations on the control of Sosnowski hogweed using a device (horn antenna) emitting microwaves at 2.45 GHz, 32.8 kW/m^2^; (2) Evaluate the impact of microwave treatment (MWT) on soil by an ecotoxicological assessment; (3) Analyze biochemical changes occurring in the roots during the process.

## Materials and methods

### Study site

The research was carried out on the population of Sosnowski's hogweed (*Heracleum sosnowskyi* Manden.), located in the western part of the Wielkie Błoto PLH 120080 peat bog (50 101132600 N 20 1505348100E; 195.6 m a.s.l.), in the Niepołomice Forest, approximately 20 km southeast of Krakow. The number of hogweed specimens was 1–3 per 1 m^2^ in a total area of 1500 m^2^. On the eastern side, it was bordered by a post-exploitation reservoir in the phase of overgrowth, with reed vegetation belonging to the *Phragmitetea* class and Canadian goldenrod (*Solidago canadensis* L.).

Soil samples were taken from the experimental site for analyses in the accredited laboratory of AgroEkspert Polska according to the following research methods: granulometric composition PN-R-04032:1998 (for samples with organic matter content < 5%); total nitrogen Kjeldahl method; pH PN-ISO 10390:1997; absorbable P_2_O_5_ PN-R-04023:1996; K_2_O PB-1 ed. of February 20, 2013; Mg PN-R-04020:1994 + Az1:2004; Mn PN93/R-04019 (1 M HCl); Zn PN92/R-04016 (1 M HCl); Cu PN92/R-04017 (1 M HCl); Fe PN-R-04021:1994 (1 M HCl); B PN93/R-04018 (1 M HCl); Cd, Ni PN-ISO 11047:2001 (Table 1).

**Table 1:** Soil characteristics at the study site.

Sand (%)	Coarse silt (%)	Fine silt (%)	pH (KCl)	Corg (%)	N (g/kg)	P_2_O_5_ (mg/100 g)	K_2_O (mg/100 g)
89	6	5	7.1	1.8	10.1	6.7	14.8

Mg (mg/100 g)	Mn (mg/kg)	Zn (mg/kg)	Cu (mg/kg)	Fe (mg/kg)	B (mg/kg)	Cd (mg/kg)	Ni (mg/kg)
> 15.0	535.7	> 50.0	6.3	3425.7	0.9	< 5.0	15.0

The field studies and collection of plant material comply with the IUCN Policy Statement on Research Involving Species at Risk of Extinction^40^.

### Microwave treatments

**Figure 1:**
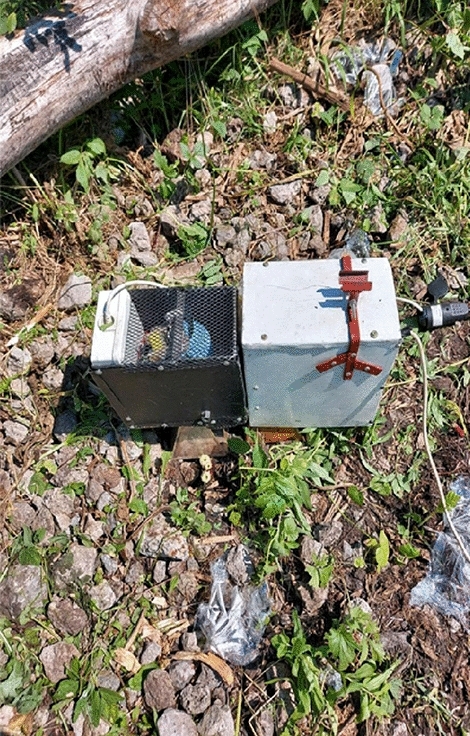
Microwave control of Sosnowski hogweed using the horn antenna. Photo, B. Grygierzec.

Fieldwork was carried out in the morning on July 14–15, 2022, when the air temperature was around 25–28 °C, air humidity 85–88%, and soil moisture 89%. Hogweed plants, approx. 60–80 cm were first cut to a height of 4–5 cm from the soil surface. Then, the cut plants were exposed to microwave radiation emitted by a horn antenna (bottom width 134 mm and length 181 mm), made of a brass sheet of 1 mm thickness, covered inside with silver and a thin layer of gold. The average radiation of the antenna is 2.45 GHz, equal to 32.8 kW/m^2^ (Fig. 1).

**Figure 2:**
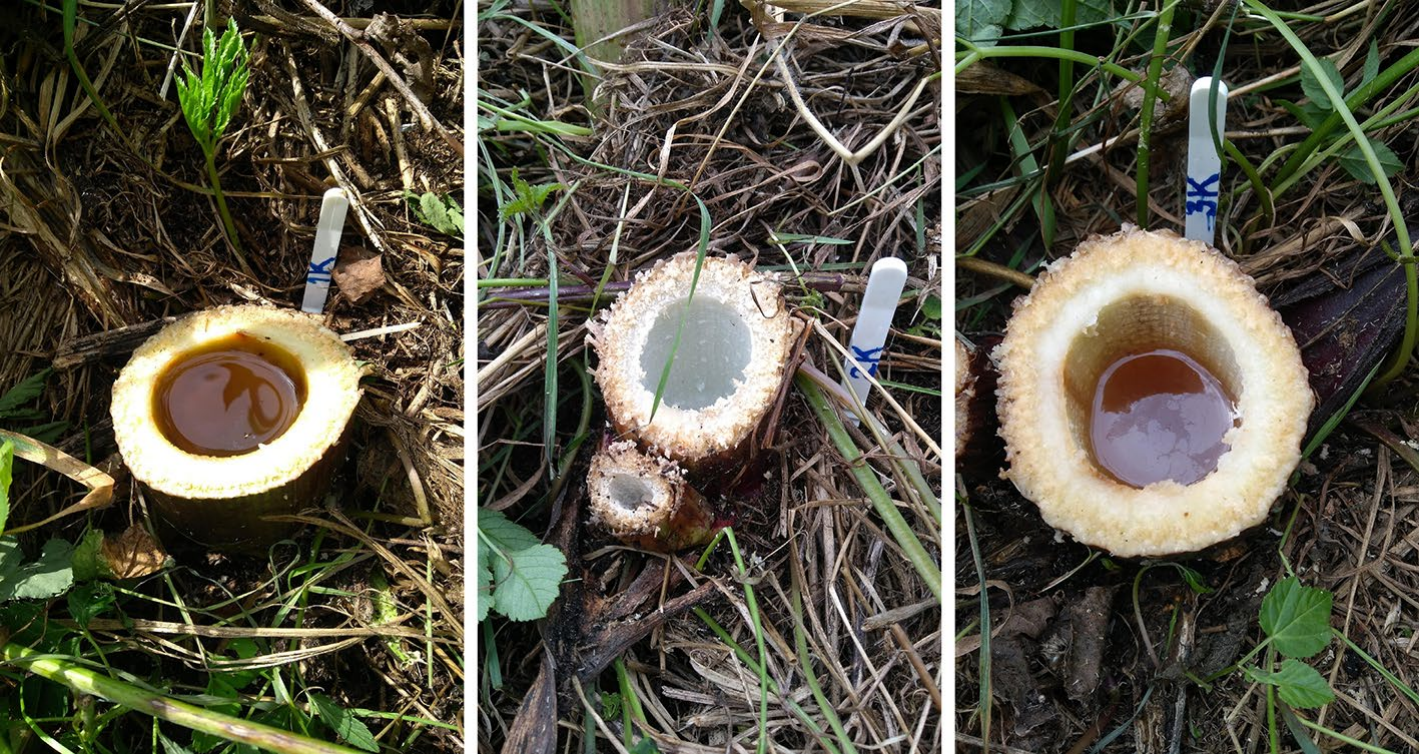
Selected control stems of Sosnowski hogweed. Photo B. Grygierzec.

Different amounts of energy reach the soil surface from the horn antenna—the most in the center and the least on the edges, as determined by the dimensions of the horn. The temperature of the substrate also changes according to the amount of energy received. The antenna adhered centrally onto the soil above each microwave-treated cut hogweed plant (MWT). The plants were MWT for 2.5, 5.0, 7.5, 10.0, 12.5, or 15.0 min. Each MWT time was performed in 6 repetitions (6 plants). Cut control plants were left without MWT (Fig. 2).

**Figure 3:**
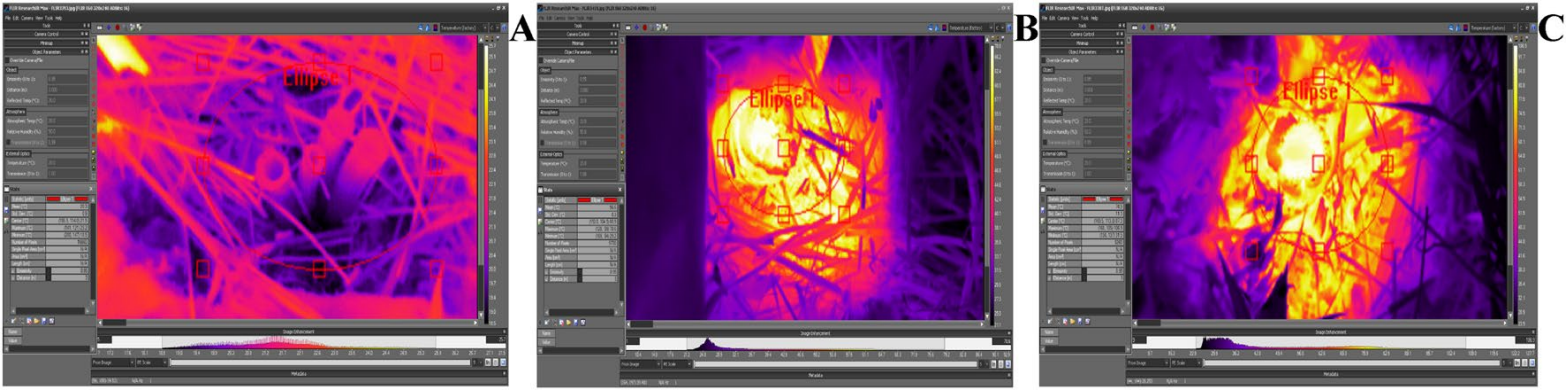
Thermograms indicate the temperature of Sosnowski hogweed stems and surrounding soil at (**A**) 0 min (control), (**B**) 7.5 min, and (**C**) 12.5 min after microwave treatment. Photo B. Grygierzec.

Before and after MWT, the temperature of the plants was measured using a FLIR E60 thermal imaging camera equipped with Wi-Fi (manufacturer: FLIR Systems, Inc., Wilsonville, Oregon, USA). The camera measures the temperature range from 20 to + 120 °C (± 2 °C) (Fig. 3). The camera was placed approximately 0.5–1 m above the MWT plant stems, and photos were taken with a resolution of 320 × 240 pixels. Each image was then analyzed using FLIR ResearchIR MAX (manufacturer: Teledyne FLIR LLC, Wilsonville, OR, USA) based on mathematical processing of the pixel color scale.

Fourteen days (July 29, 2022) after hogweed's microwave treatment (MWT), the 25–40 cm-long plant roots were dug out from the soil. The collected roots were cleaned and air-dried in paper bags in a shaded and airy place.

After 18 days of drying (August 16, 2022), the hogweed roots were thoroughly cleaned to remove any soil residue and weighed on a laboratory scale with an accuracy of 0.01 g.

#### Soil ecotoxicity

Soil ecotoxicity was evaluated through three biotests: Phytotoxkit, Ostracodtoxkit, and Microtox (Table 2). Phytotoxkit evaluated germination inhibition (%) and root growth inhibition (%) of *Sorghum saccharatum* and *Sinapis alba* after three days of soil incubation^41^. Ostracodtoxkit tested mortality and growth inhibition of *Heterocypris incongruens* after six days of incubation^42^. Microtox measured the luminescence of *Alivibrio fischeri* bacteria^43^. Soil samples were tested through the Screening Test at 81.9% using the M 500 Analyzer and classified as non-toxic (< 20%), low-toxic (20–50%), or toxic (50–100%).

**Table 2:** Characteristics of biotests battery.

Biotest	Test organism	Final parameter	Biotest duration
Phytotoxkit	*Sinapis alba* *Sorghum accharatum*	Inhibition of germination, inhibition of radicle growth	72 h
Ostracodtoxkit	*Heterocypris incongruens*	Death, inhibition of growth	6 days
Microtox	*Alivibrio fischeri*	Inhibition of luminescence	15 min

### Isolation of volatile compounds in the microwave-treated Sosnowski hogweed roots

Volatile compounds from crushed roots, including the volatile photosensitizing chemicals, were hydrodistilled as essential oils (EOs) for 4 h using a Clevenger-type apparatus. Hydrodistillation was performed once for each sample until the volume of essential oil did not increase in the glass tube of the Clevenger apparatus. GC-FID-MS identified and quantified the essential oils' components with a quadruple MS detector and Advanced Ion Source for the electron impact (EI). GC Ultra coupled with a DSQII mass spectrometer (Thermo Electron). Simultaneous GC-FID and GC–MS analysis was performed using an MS-FID splitter (SGE Analytical Science). Mass spectra in the positive ions mode were recorded in the m/z 50–550 range after EI ionization at 70 eV. Operating conditions: capillary column Rtx-1 MS (60 m × 0.25 mm i.d., film thickness 0.25 μm), and temperature program: 50 (3 min)–300 °C (30 min) at 4 °C/min. Injector and detector temperatures were 280 °C and 300 °C, respectively. Carrier gas was helium (constant pressure: 300 kPa). Compound identification was based on comparing mass spectra with the computer mass library NIST 98.1, Wiley Registry of Mass Spectral Data, 10^th^ edition, and relative retention indices (RI, on non-polar column).

### Sugar composition and content

The glucose, fructose, saccharose, and maltose content were measured in the control and MWT plant tissues. Samples of plant tissues were lyophilized and then homogenized. Aliquots of approximately 50 mg of samples were extracted in 1 ml of 70% aqueous ethanol by sonication for 30 min and centrifuged for 10 min at 10 °C at 39 000 *g* (Universal 32R, Hettich, Germany). The solution was evaporated to dryness under a stream of nitrogen gas and then dissolved in 1 ml of pure water. The dissolution process was assisted by sonication for 30 min. Then, the samples were centrifuged and placed in HPLC vials.

HPLC analyses were performed using an Agilent Technologies 1200 chromatograph (Agilent, USA) with an ESA Coulochem II amperometric detector with an analytical cell type 5040, using an RCX-10 (250 × 4.1 mm, 5 μm) chromatography column (Hamilton, USA). The mobile phases (A) 80 mM sodium hydroxide solution and (B) 80 mM sodium hydroxide solution with 500 mM sodium acetate were used, with a gradient elution of 1.5 ml/min at 40 °C. The amperometric detector operated in pulse mode with potentials: analytical potential 200 mV, 500 ms; oxidizing potential 700 mV, 100 ms; reducing potential − 900 mV, 100 ms. A cleaning cell (Guard cell) before the autosampler unit, with a potential of 250 mV, was also used. The injection volume was 0.01 ml. The analysis time was 20 min, and six biological replications were performed for each treatment.

### Lipid extraction and fatty acid composition

Fifty mg samples of lyophilized and homogenized plant tissues were extracted in 1 mL of chloroform for 1 h in a mixing mill at a frequency of 30 Hz, then sonicated for 15 min and centrifuged for 5 min at 22 000 g (Universal 32R, Hettich, Germany), supernatants were collected. The extraction procedure was repeated twice, and the final supernatants were combined. Next, 2 mL of concentrated sulfuric acid (95–97%) in methanol (1:4) was added to the supernatant and heated in a heating block for 1 h at 100 °C, shaking every 20 min. After cooling, 1 mL of H_2_O was added and shaken vigorously. After phase separation, 1 mL was taken from the lower chloroform phase, filtered through a cotton wool filter directly into vials, and kept at − 20 °C until analysis. The analyses were performed in six biological replicates.

The composition of fatty acids (FA) was analyzed using gas chromatography (GC). The samples were analyzed using an Agilent Technologies 7820A (Santa Clara, CA, USA) gas chromatograph equipped with a flame-ionization detector (FID) and an HP-88 60 m × 250 m × 0.2 m GC column (Agilent Technologies, Santa Clara, CA, USA). The inlet temperature was set to 280 °C with a split mode (10:1). The mobile phase was hydrogen, and gas flow was set to 2 mL/min. The temperature gradient that was used was as follows: initial 120 °C hold 1 min, ramp 10 °C/min to 175 °C, hold 8 min, ramp 5 °C/min to 210 °C, hold 3 min, and finally, post time 2 min with 250 °C. The detector temperature was set to 280 °C. The individual fatty acid methyl esters were identified by comparing their retention times with a standard mixture of Supelco 37 component FAME Mix (Merck, Darmstadt, Germany).

The data were expressed as the molar percentage of a specific fatty acid relative to all the measured fatty acids.

### Statistical analysis

The normality of the distributions of the observed traits was tested using Shapiro–Wilk's normality test^44^. The roots' air-dry mass and ecotoxicological biotests' results were analyzed statistically using a one-way analysis of variance (ANOVA) for a completely randomized design. Since the data was non-normally distributed, logarithmized data was used for analysis. The HSD Tukey post-hoc test was used to determine the differences between means' significance.

One-way ANOVA was carried out to determine the effects of a group of essential oil compounds (aliphatic aldehydes and ketones, aliphatic alcohols and esters, monoterpene hydrocarbons, oxygenated derivatives of monoterpene hydrocarbons, sesquiterpene hydrocarbons, oxygenated derivatives of sesquiterpene hydrocarbons, compounds based on p-isopropylmethylbenzene skeleton, structurally similar active compounds, lactones, furans and furanocoumarins, others) on the variability of the observed traits. The mean values and standard deviations of traits were calculated. Moreover, Fisher's least significant differences (LSDs), at the 0.05 level, were calculated, and on this basis, homogeneous groups were determined for the groups of compounds. The relationships between observed traits were estimated using Pearson's linear correlation coefficients and are presented in a heatmap. The results were also analyzed using multivariate methods. A canonical variance analysis (CVA) was applied to present a multi-trait assessment of the similarity for the tested groups of compounds in lower dimensions with the least possible loss of information. The Mahalanobis distances^45^ were used as a measure of the "multi-trait" similarity of groups of compounds^46^, the significance of which was verified using the critical value *D*_*a*_, the least significant distance^47^. Mahalanobis distances were calculated for all pairs of groups of compounds.

The multifactorial ANOVA and Duncan test at a significance level of *p* ≤ 0.05 were used to analyze sugar and fatty acids content.

The GenStat v. 23.1 statistical software package (VSN International Genstat for Windows, 2023; vsni.co.uk/software/genstat) and Statistica 13.3 software (Tibco Software, USA; www.tibco.com) were used for the analyses.

## Results

### Soil ecotoxicity

In the Phytotoxkit test, inhibition germination of *S. alba* ranged from 0 to 21% (Table 3). The highest germination inhibition was demonstrated for soil exposed to microwaves for 7.5 and 12.5 min. The inhibition root growth of *S. alba* ranged from − 23 to 49%. The greatest inhibition of root growth was demonstrated in the soil exposed to 12.5 min of microwave. In soil exposed to microwaves for 2.5 min, 5 min, and 15 min, stimulation of the growth of the roots of *S. alba* was found. The remaining samples showed low toxicity to *S. alba*. The soil exposed to microwaves was found to be less toxic to *S. saccharatum* compared to *S. alba*. Inhibition of *S. saccharatum* germination was 0–17%, and root growth inhibition ranged from − 13 to 15%. Stimulation of *S. saccharatum* root growth was demonstrated in the control soil and after 5 and 15 min exposure to microwaves. The remaining soil samples showed minimal inhibition of root growth. The greatest growth stimulation was found in the soil exposed to microwaves at 15 min, and the greatest inhibition in the soil exposed at 10 min. Generally, the analyzed soil samples were non-toxic to *S. saccharatum*.

**Table 3:** Ecotoxicity of the soil in the proximity of Sosnowski hogweed plants treated with microwaves for 0–15.0 min in a field experiment.

Soil exposed to microwaves (mins)	*Sinapis alba*		*Sorghum saccharatum*		*Heterocypris incongruens*		*Alivibrio fischeri*	Toxicity class
	GI*	RGI	GI	RGI	M	LI	ILU	
	Percentage toxicity effect PE%							
0	14 ab**	14	17 b	− 5	0	19** b	32** d	II
2.5	0 a	− 23	0 a	11	0	− 11 a	27 c	I
5.0	14 ab	− 7	0 a	− 2	0	38 c	20 ab	II
7.5	21 b	23	8 ab	9	0	6 ab	22 b	II
10.0	14 ab	29	0 a	15	0	15 ab	16 a	II
12.5	21 b	49	8 ab	3	0	− 18 a	19 ab	II
15.0	0 a	− 9	0 a	− 13	0	15 ab	22 b	I

*GI—germination inhibition, RGI—root growth inhibition, M—mortality, LI—length inhibition, ILU—luminescence inhibition.

**Numbers followed by different letters indicate statistically significant differences.

In the Ostracodtoxkit test, crustacean (*H. incongruens*) growth inhibition ranged from − 18 to 38% (Table 3). The greatest inhibition of crustacean growth was demonstrated in the soil exposed to microwaves for 5 min. Crustacean growth was stimulated in the soils exposed for 2.5 min and 12.5 min. Most microwave-exposed soils were non-toxic to crustacean. Only soil exposed for 5 min was classified as low-toxic.

Inhibition of *A. fischeri* luminescence ranged from 16 to 32% (Table 3). The highest toxicity towards bacteria was observed in the control and lowest in the soil exposed to microwaves for 10 and 12.5 min. Soils exposed for 10 min and 12.5 min were non-toxic to *A. fischeri*. The remaining soils were characterized by low toxicity to bacteria.

According to the hazard classification (Table 3), considering the response of all organisms, soil treated with microwaves for 2.5 and 15 min was classified as class I toxicity, which means that these samples are non-toxic. The remaining soils are classified as class II, meaning they are low-toxic and do not pose a significant risk to living organisms.

### Thermograms

The average temperature of control, non-MWT, plants was 20.3 °C, while for the MWT plants at times 5.0, 7.5, 10.0, 12.5, 15.0, 20, and 25.0 min reached the following temperatures: 35.2, 42.0, 56.6, 66.5, 74.5, 84.5 °C (Table 4).

**Table 4:** The mean (± standard deviation), maximum, and minimum temperatures (°C) of Sosnowski hogweed plants microwave-treated for 0–15.0 min in a field experiment.

Treatment duration (mins)	0.0	2.5	5.0	7.5	10.0	12.5	15.0
Mean temperature	20.3 ± 0.9	35.2 ± 2.3	42.0 ± 3.9	56.6 ± 8.3	66.5 ± 10.2	74.5 ± 11.3	84.5 ± 31.6
Maximum temperature	23.2	42.4	52.1	78.6	92.4	106.9	> 150.2
Minimum temperature	18.5	29.0	31.6	29.2	35.9	31.5	41.4

The values are based on the FLIR E60 thermal imaging camera readings.

### Sosnowski hogweed roots

**Figure 4:**
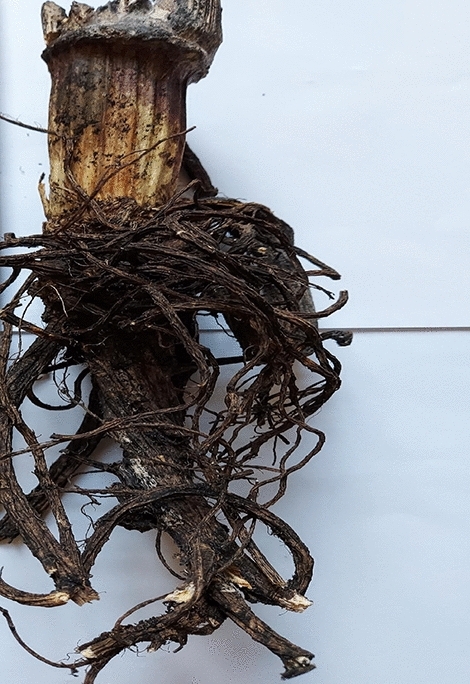
Example of air-dried Sosnowski hogweed control roots, not microwave-treated. Photo B. Grygierzec.

**Figure 5:**
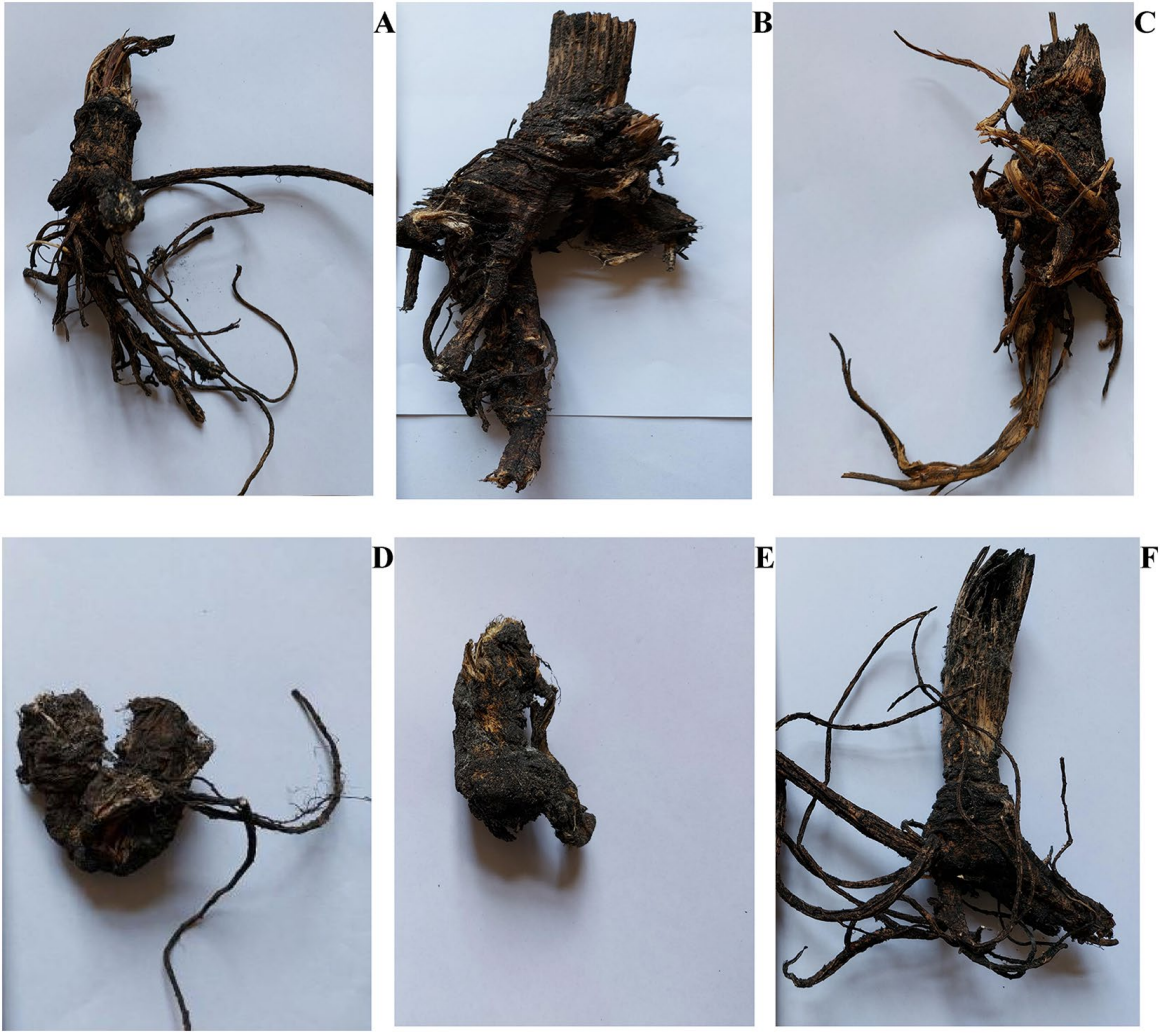
**A–F** Examples of air-dried Sosnowski hogweed roots microwave-treated at times: (**A**) 2.5 min, (**B**) 5.0 min, (**C**) 7.5 min, (**D**) 10.0 min., (**E**) 12.5 min, (**F**) 15.0 min. Photo B. Grygierzec.

Figures 4 and 5 present the examples of S. hogweed dry roots, 14 days after MWT.

The roots of Sosnowski's hogweed were air-dried for 18 days, and their weights were measured (Table 5). The ANOVA analysis revealed that the differences in root biomass based on MWT times were statistically insignificant due to internal differences between root mass within one treatment. The control roots had the highest biomass (average 101.0 g), while the roots MWT for 12.5 min had the lowest biomass (average 56.3 g). The average weight of MWT roots for 15.0 min was 69.3 g and was *ca*. two times smaller than the mass of control roots. There was no visible trend in root biomass change between the MWT plants.

**Table 5:** Average weight (g) of air-dried Sosnowski hogweed roots.

Microwave treatment time (mins)	Mass of air-dry roots (g)	SE
0 (control)	101.0	16.8
2.5	64.7	10.8
5.0	84.6	14.1
7.5	85.8	14.3
10.0	87.8	14.6
12.5	56.3	9.4
15.0	69.3	11.6

The table shows the mean values and standard error (n = 6).

SE—standard error, F—Fisher test value, F = 1.75. Statistical analysis was performed for logarithmized data and *p*—significance level, *p* = 0.14.

#### Analysis of volatile compounds in Sosnowski hogweed roots after microwave treatment

The essential oil containing the volatile components of S. hogweed roots was obtained by hydrodistillation, which lasted for several hours. The oil had a strong herbal aroma and an efficiency range of 0.1–0.2%. The data showed that MWT duration did not affect essential oil yield (Supplementary Table 1).

Significant qualitative differences were noticed among the more than twenty tested oils extracted from the plant's roots. Supplementary Table 1 contains the average values of the content of volatile components identified in the tested objects. A total of 90 volatile compounds were identified in the oils. They constituted 85–91% of all isolated essential oil components. Average values for oils marked as an essential oil (EO) A, B, and C were obtained based on analyses of the efficiency and composition of 3 essential oils for each group, oils marked as EO D, E, and F—based on the average of 5 oils for each of the groups (Supplementary Table 1). In the upper part of the table, essential oils from groups A to F are assigned to the appropriate time of exposure to microwaves: 2.5, 5.0, 7.5, 10.0, 12.5, and 15 min.

The results of GC–MS analyses revealed that the main component of all OE from hogweed roots microwave-treated was p-cymene, constituting from 11.9 ± 11.5% of oil treated for 2.5 min (EO A) to 40.1 ± 14.9% in the oil (EO F) treated for 15 min.

Among the furanocoumarins, angelicin and 6,7-dimethoxyangelicin were identified. The content for angelicin was determined in EO C, D, and E at a low level of 0.1–0.2%. The anglicin molecule enriched with two ethoxyl groups was identified at 0 ± 0 to 1.4 ± 1.8%. There is no relationship between the exposure time of hogweed roots to microwaves and the change in the content of these compounds in the roots' oil.

It is worth noting that, apart from p-cymene, the other dominant OE components from hogweed roots are γ-palmitolactone, which contains a saturated, oxidized, and substituted furan ring. The furan ring, in turn, is a characteristic fragment of the structures of furanocoumarins, which is considered photosensitizing. The presence of γ-palmitolactone was determined from 0 ± 0.1 to 21.4 ± 33.6% in EO F and EO C, respectively. In oils from the EO C group, obtained from roots MWT for 7.5 min, this compound in one of the five oil samples accounted for more than 50%. Other components of root EO containing the furan ring are, for example, 2-pentylfuran, 3,6-dimethyl-2,3,3α,4,5,7α-hexahydrobenzofuran and alantolactone. However, these components constitute a negligible percentage of the root oil.

Hogweed roots contain many aliphatic esters and aliphatic alcohols responsible for the plant's potential photosensitizing properties. Thus, the ingredients that particularly stand out in EO from Sosnowski's hogweed roots are heptadec-(9Z)-ene-4,6-diyn-8-ol and (Z)-heptadeca-1,9-diene-4,6- diyne-3-ol or falcarinol. These are aliphatic alcohols, the average content of which in the tested EO ranged from 3.3 ± 3.9 to 13.8 ± 18.9% for the first alcohol and from 1.3 ± 1.2 to 5.1 ± 7.8% for falcarinol. The content of the above two unsaturated aliphatic alcohols in some EO samples exceeded 30% of the oil (one of the EO C samples).

One of the ingredients of oils of strong biological properties is myristicin. Its content in the EOs is noticeable and ranges from 1.2 ± 0.5 to 8.1 ± 6.5%, which means that in one of the batches of raw material from which oils marked as EO F were obtained, the myristicin content was close to 15%.

The statistical analysis of individual components was numerically complex, so it did not answer our questions. Hence, we formed clusters of compounds of structural similarity from nearly 100 compounds identified in the oils. Analysis of variance indicated that the main effects of a group of compounds were significant for the sum of monoterpene hydrocarbons (MT) (Table 6). The highest value of MT sum was observed for the control EO (no MWT), 38.4%. For the EO F of the longest time that microwaves were used on the raw material (15 min), the value of this feature was 11.78%, and it was a separate homogeneous group. No statistically significant differences were observed between the other times of microwave treatment on the raw material (EO A, EO B, EO C, EO D, and EO E), and the sum of MT ranged from 4.83% (for EO A0 to 9.31% (for EO E) (Table 6).

**Table 6:** Results of the analysis of variance for the main chemical groups of essential oil components from hogweed roots.

EOs	AA&K	AA&E	MT	MTO	ST	STO	IPB	SAC	Lactones	F&F	Others
EO	0.2 ± 0	1.6 ± 0.3	38.4 ± 0.6	0.1 ± 0.1	1.6 ± 0.1	0.8 ± 0.0	56.1 ± 0.6	0 ± 0	0 ± 0	0.4 ± 0.03	0.1 ± 0
EO A	2.3 ± 1.3	9.5 ± 4.7	4.8 ± 4.2	3.5 ± 2.6	3.3 ± 2.1	16.8 ± 13.3	16.1 ± 12	14.1 ± 10.6	0.5 ± 0.36	10.3 ± 11.3	4.2 ± 2.9
EO B	1.5 ± 0.5	5.2 ± 4.5	8.5 ± 7.3	1.0 ± 1.1	0.9 ± 0.6	14.2 ± 6.02	17.6 ± 15.4	15.7 ± 9.1	0.3 ± 0.4	9.0 ± 7.1	5.3 ± 6.6
EO C	0.5 ± 0.7	16.1 ± 19.1	7.9 ± 9.9	0.8 ± 0.5	1.5 ± 1.4	5.8 ± 1.4	22.9 ± 19.9	3.3 ± 0.8	0.1 ± 0.2	21.7 ± 3.9	3.4 ± 2.6
EO D	3.0 ± 2.4	9.4 ± 9.7	8.8 ± 11.2	4.2 ± 2.9	3.9 ± 2.8	13.8 ± 9.2	28.8 ± 22.9	6.5 ± 6.9	0.2 ± 0.3	8.2 ± 1.1	1.8 ± 1.4
EO E	3.6 ± 2.7	15.3 ± 26.4	9.3 ± 8.4	1.1 ± 1.1	1.9 ± 2.7	7.9 ± 10.6	34.3 ± 28.9	11.9 ± 20.6	0 ± 0	5.2 ± 8.4	0.6 ± 0.8
EO F	1.4 ± 1.2	5.4 ± 5.3	11.8 ± 3.5	2.1 ± 1.8	1.1 ± 1.87	4.7 ± 4.8	40.8 ± 14.4	19.7 ± 11.2	0 ± 0	1.7 ± 2.01	0.4 ± 0.8
*F*-ANOVA	0.16	0.86	0.005	0.07	0.41	0.23	0.33	0.43	0.06	0.52	0.09
LSD_0.05_	3.2	26.3	13.5	3.1	3.7	14.3	35.4	21.6	0.4	22.5	4.4

The components are expressed in the essential oil content percentage (± standard deviation).

AA&K—aliphatic aldehydes & ketones; AA&E—aliphatic alcohols and esters; MT—monoterpene hydrocarbons; MTO—oxygenated derivatives of MT; ST—sesquiterpene hydrocarbons; STO—oxygenated derivatives of ST; IPB—Compounds based on the p-isopropylmethylbenzene skeleton (p-cymene, thymol and carvacrol and their methyl esters etc.); SAC—structurally similar active compounds, i.e., miristicine, asarone and eugenol with their derivatives, elemicine; Lactones; F&F—furans and furanocoumarins; Others.

**Figure 6:**
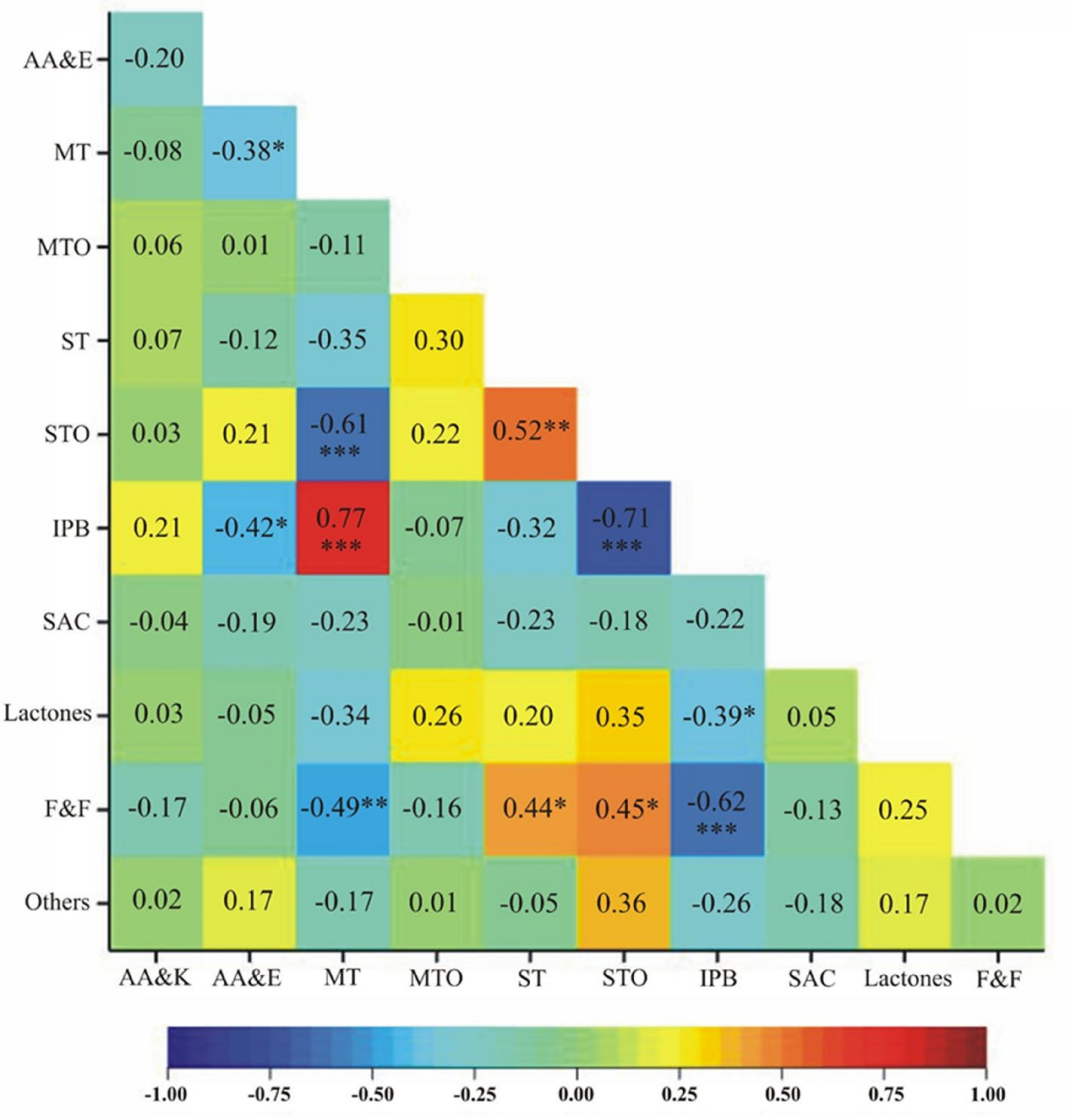
Correlation matrix for the groups of compounds of essential oils from *Heracleum Sosnowskyi* roots. AA&K—aliphatic aldehydes & ketones; AA&E—aliphatic alcohols and esters; MT—monoterpene hydrocarbons; MTO—oxygenated derivatives of MT; ST—sesquiterpene hydrocarbons; STO—oxygenated derivatives of ST; IPB—Compounds based on the p-isopropylmethylbenzene skeleton (p-cymene, thymol and carvacrol and their methyl esters etc.); SAC—structurally similar active compounds, i.e., miristicine, asarone and eugenol with their derivatives, elemicine; Lactones; F&F—furans and furanocoumarins; Others.

Statistically significant correlations were observed for 11 pairs of observed traits. A positive correlation was characterized by pairs of traits: sum of STO—a sum of ST (0.5), a sum of F&F—a sum of ST (0.4), a sum of IPB—a sum of MT (0.8), and a sum of F&F—a sum of STO (0.4). In contrast, statistically significant negative correlation coefficients were observed for a sum of MT—a sum of AA&E (− 0.4), a sum of IPB—a sum of AA&E (− 0.4), a sum of STO—a sum of MT (− 0.6), a sum of F&F—a sum of MT (− 0.5), a sum of IPB—a sum of STO (− 0.7), a sum of lactones—sum of IPB (− 0.4), and sum of F&F—sum of IPB (− 0.6) (Fig. 6).

**Figure 7:**
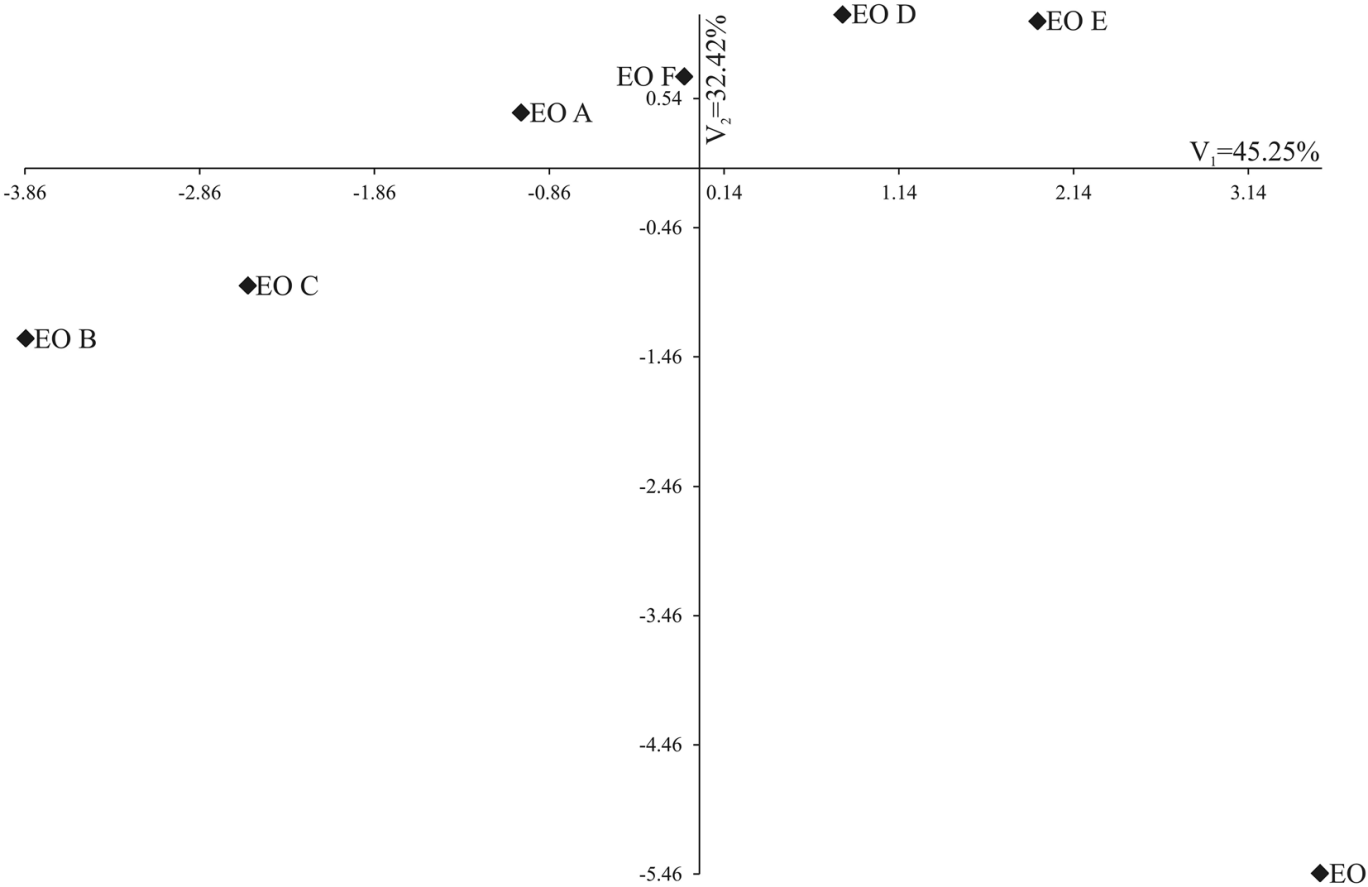
The distribution of essential oils of *H. Sosnowskyi* in the two first canonical variables. In the diagram, the coordinates of a given combination of treatments are values of the first and second canonical variables.

Samples from EO A to EO F and the control sample (EO) were also compared based on all observed traits. For this purpose, a canonical variable analysis was used (Table 7), and Mahalanobis distances (Table 8) were used. The first two (V_1_ and V_2_) canonical variables accounted for 77.67% of the total sample variability (Fig. 7). The V_1_ was significantly positively correlated with the sum of compounds based on the p-isopropylmethylbenzene skeleton (IPB) (0.828) and negatively with the sum of Others (− 0.872). The V_2_ was positively correlated with the sum of aliphatic aldehydes & ketones (AA&K) (0.768) and negatively correlated with the sum of monoterpene hydrocarbons (MT) (− 0.880) (Table 7). The greatest variation of all the eleven traits jointly measured with Mahalanobis distances was found for the EO (control) and EO B (MWT for 5 min) (= 8.56) and EO and EO C (MWT for 7.5 min) (= 8.02). The greatest similarity (= 2.35) was found between EO D (MWT for 10.0 min) and EO E (MWT for 12.5 min) (Table 8).

**Table 7:** Analysis of the first two canonical variables for the main groups of compounds of essential oils from *Heracleum Sosnowskyi* roots.

	V_1_	V_2_
AA&K	0.149	0.768*
AA&E	− 0.216	0.581
MT	0.67	− 0.880**
MTO	− 0.053	0.67
ST	0.233	0.401
STO	− 0.514	0.552
IPB	0.828*	− 0.552
SAC	− 0.405	0.603
Lactones	− 0.594	0.213
F&F	− 0.673	0.219
Others	− 0.872*	0.131

AA&K—aliphatic aldehydes & ketones; AA&E—aliphatic alcohols and esters; MT—monoterpene hydrocarbons; MTO—oxygenated derivatives of MT; ST—sesquiterpene hydrocarbons; STO—oxygenated derivatives of ST; IPB—Compounds based on the p-isopropylmethylbenzene skeleton (p-cymene, thymol and carvacrol and their methyl esters etc.); SAC—structurally similar active compounds, i.e., miristicine, asarone and eugenol with their derivatives, elemicine; Lactones; F&F—furans and furanocoumarins; Others.

*p < 0.05; **p < 0.01

**Table 8:** Mahalanobis distances for the analysed essential oils from *Heracleum Sosnowskyi* roots.

	EO	EO A	EO B	EO C	EO D	EO E	EO F
EO	0						
EO A	7.69	0					
EO B	8.56	4.02	0				
EO C	8.02	4.41	3.51	0			
EO D	7.26	2.63	5.59	4.88	0		
EO E	6.91	4.14	6.46	5.34	2.35	0	
EO F	7.35	3.51	4.71	3.99	2.74	2.74	0

Essential oils from plants: EO—control (non-microwave treated); EO A—microwave treated for 2.5 min; EO B—microwave treated for 5.0 min; EO C—microwave treated for 7.5 min; EO D—microwave treated for 10 min; EO E—microwave treated for 12.5 min; EO F—microwave treated for 15 min.

Essential oils from plants microwave treated for EO—control 0 min; EO A—2.5 min; EO B—5.0 min; EO C—7.5 min; EO D—10 min; EO E—12.5 min; EO F—15 min.

#### Sugar composition and content

**Figure 8:**
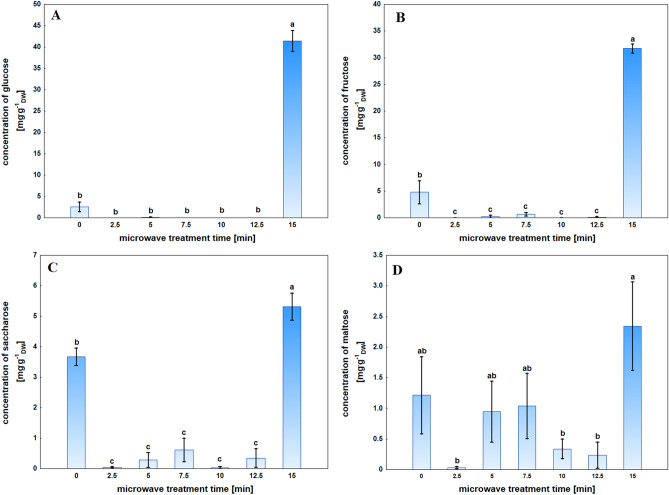
Content of sugars: glucose (**A**), fructose (**B**), saccharose (**C**), and maltose (**D**) in Sosnowski hogweed tissue of control and microwave-treated for a specified time. Mean values ± SD marked with the same letters do not differ significantly at *p* ≤ 0.05 according to Duncan's test, n = 6.

The highest glucose content was observed in plant tissue after 15 min of MWT. There was a 16-fold increase compared to the control, which was a statistically significant difference. For the remaining MWT times, the glucose content was almost zero (Fig. 8A).

The highest concentration of fructose was also recorded for samples exposed to MWT for 15 min. A statistically significant increase of over tenfold was observed compared to the control. MWT for 2.5–12.5 min resulted in a statistically significant decrease in the fructose content in the analyzed samples (Fig. 8B).

A 15 min MWT resulted in a statistically significant increase in saccharose concentration by approximately 44% compared to the control. However, the remaining MWT time caused a significant decrease in saccharose content by 83% for samples MWT for 7.5 min and approximately 99% for samples MWT for 2.5 min and 10 min compared to the control (Fig. 8C).

The concentration of maltose was the highest for the plants that were exposed to MWT for 15 min, and it was almost a twofold increase compared to the control. After 2.5, 10, and 12.5 min, MWT caused a statistically significant decrease of almost 99%, over 85% and 90%, respectively, compared to the 15 min exposed plants. Maltose concentrations for plants exposed for 5 and 7.5 min were similar to control samples (Fig. 8D).

#### Fatty acids (FA) composition and content

The following fatty acids were found in Sosnowski's hogweed tissue: 12:0 (laureic), 14:0 (myristic), 15:0 (pentadecanoic), 16:0 (palmitic), 16:1 (palmitoleic), 18:0 (stearic), 18:1 (oleic), 18:2 (linoleic), 18:3 (α-linolenic and g-linolenic) and 22:0 (behenic) (Table 9). According to the accepted notation, the first number indicates the number of carbon atoms. In contrast, the number after the colon indicates the number of double bonds in the fatty acid molecule.

**Table 9:** Composition of fatty acids (molar %) in Sosnowski hogweed root tissue (control and microwave-treated for a specified time).

MWT (mins)	12:0	14:0	15:0	16:0	16:1	18:0	18:1	18:2	g-18:3	α-18:3	22:0	non—identified
0	10.51 ± 2.71b	1.37 ± 0.39d	1.13 ± 0.25a	30.28 ± 2.44c	3.21 ± 1.09c	6.76 ± 1.09bc	2.04 ± 0.43b	35.38 ± 1.45a	1.29 ± 0.23ab	1.75 ± 0.49c	2.38 ± 0.66a	3.90
2.5	13.74 ± 1.85a	3.37 ± 1.64ab	1.21 ± 0.36a	30.08 ± 5.07c	4.52 ± 1.35b	6.17 ± 1.37c	4.45 ± 0.83a	27.24 ± 1.84bc	1.06 ± 0.39b	2.53 ± 0.71a	1.93 ± 0.65a	3.70
5	11.85 ± 1.49ab	2.03 ± 0.80bcd	1.62 ± 0.48a	41.45 ± 5.02a	2.26 ± 0.87c	7.79 ± 1.93ab	2.09 ± 0.65b	22.30 ± 3.00d	1.73 ± 0.44a	2.27 ± 0.86abc	1.79 ± 0.56a	2.82
7.5	10.37 ± 0.85b	2.55 ± 0.88abcd	1.21 ± 0.25a	37.13 ± 1.89b	2.03 ± 0.93c	9.00 ± 3.00a	1.74 ± 0.34b	20.04 ± 2.85d	0.99 ± 0.21b	2.58 ± 0.83abc	1.67 ± 0.68a	10.69
10	12.23 ± 1.11ab	3.77 ± 1.07a	1.53 ± 0.56a	32.54 ± 4.01bc	8.14 ± 1.77a	6.54 ± 0.96bc	3.79 ± 0.89a	20.10 ± 3.52d	0.99 ± 0.19b	2.84 ± 0.66ab	2.65 ± 0.44a	4.88
12.5	11.55 ± 2.38ab	2.94 ± 1.04abc	1.40 ± 0.29a	32.36 ± 2.67bc	5.43 ± 1.49b	6.73 ± 1.11bc	3.41 ± 1.10a	25.53 ± 6.62cd	1.02 ± 0.10b	2.87 ± 0.47ab	2.22 ± 0.73a	4.54
15	10.86 ± 2.35b	1.60 ± 0.76cd	1.47 ± 0.37a	37.27 ± 3.00b	1.92 ± 0.41c	7.79 ± 1.64abc	1.04 ± 0.61b	29.58 ± 5.31ab	1.56 ± 0.32a	1.97 ± 0.50bc	1.96 ± 1.19a	2.98

Mean values ± SD marked with the same letters do not differ significantly at *p* ≤ 0.05 according to Duncan's test, n = 6.

Different MWT times of plant tissue did not cause statistically significant differences in the content of 15:0 and 22:0 acids. Only 2.5 min MWT significantly increased FA 12:0 concentration by over 30% compared to the control. A significant increase in FA 14:0 concentration by over twofold was observed for samples exposed to MWT for 2.5, 10 and 12.5 min compared to the control. The 16:0 acid was the most abundant among the saturated acids. A significant increase in the content of this FA was caused by 5, 7.5, and 15 min MWT by approximately 37%, 22%, and 23%, respectively. MWT for 2.5, 10, and 12.5 min resulted in a statistically significant increase in FA 16:1 and 18:1 concentrations, compared to the control plants, with the lowest concentration recorded for plants subjected to MWT for 15 min. The highest concentration of FA 18:0 and, at the same time, a statistically significant difference (over 33% increase) compared to the control was recorded only for plants subjected to MWT for 7.5 min. The concentration of FA 18:2 was the highest for the control plants. MWT from 2.5 to 12.5 min resulted in a statistically significant decrease compared to non-treated plants. No statistically significant changes were observed for FA g-18:3. However, in the case of a-18:3, the lowest value was recorded for control plants, and MWT for 2.5, 10, and 12.5 min caused statistically significant differences—an increase of over 44%, approximately 62% and 64%, respectively.

**Figure 9:**
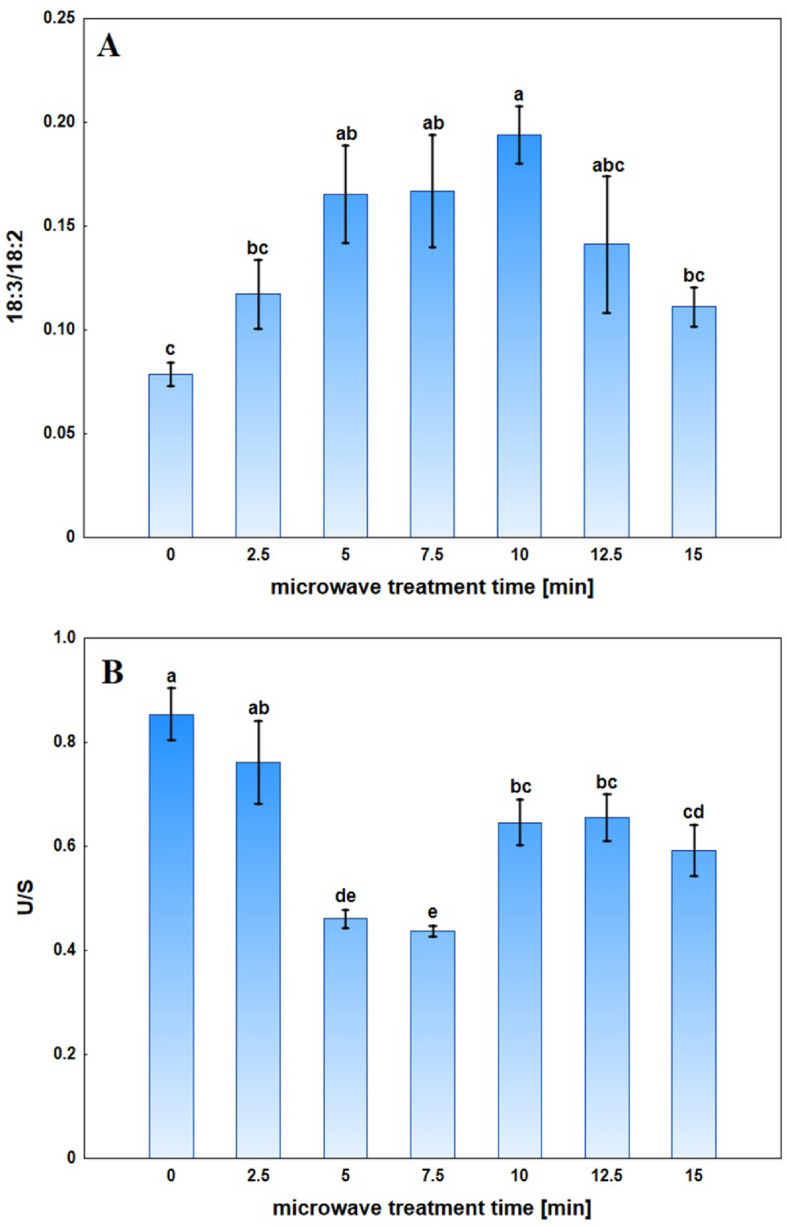
The ratio of fatty acid 18:3–18:2 (18:3/18:2)—(**A**) and unsaturated fatty acids to saturated fatty acids (U/S)—(**B**) for the Sosnowski hogweed roots tissue of control and microwave-treated for a specified time. Mean values ± SD marked with the same letters do not differ significantly at *p* ≤ 0.05 according to Duncan's test, n = 6.

The ratio of 18:3/18:2 was calculated because these two unsaturated FA are the most important for the properties of the membranes, especially concerning temperature (Fig. 9A). The lowest value of this ratio was recorded for the control plants. As the MWT time increased from 2.5 to 10 min, the value of this ratio gradually increased, followed by a decrease.

The U/S parameter (the sum of unsaturated fatty acids to the sum of saturated fatty acids) was selected to assess changes in the unsaturation of fatty acids (Fig. 9B). The highest value of this ratio was noted for the control plants, while the lowest value was recorded for plants exposed to MWT for 7.5 min. The U/S ratio was significantly lower for plants exposed to MWT from 5 to 15 min than the control.

## Discussion

Microwave treatment (MWT) effectively controlled Sosnowski hogweed, regardless of treatment time ranging from 2.5 to 15 min. The plants did not regrow 14 days after MWT, which could potentially enhance the productivity of native flora in the habitat of interest. The MWT also reduced root biomass by 55–67% in treated plants. The results point to the potential of the microwave method in hogweed control. The probable factors influencing the method's effectiveness, such as the plant age, stem diameter, and plant condition (mainly hydration), could cause the heterogenic response of the hogweed population to the MWT and should be analyzed in further studies.

The high temperature generated by MWT did not cause negative soil ecotoxicity effects. According to the hazard classification, considering the responses of all soil organisms in the ecotoxicological biotests, MWT soils were classified as not threatening the environment (class I) or low-toxic and did not threaten the environment (class II). That result is consistent with our previous results on the ecotoxicity of sandy soil near knotweed (*Reynoutria japonica*) MWT for 5–25 min^36^.

According to scientific literature, Sosnowski hogweed essential oil (EO) has been extensively researched, but only from aboveground parts of plants. The roots were not of interest to scientists. The yield of oil from S. hogweed roots was much lower than that of the seeds of this species (5.1%), according to Synowiec and Kalemba^48^. There is a significant diversity in the composition of EOs obtained from different aboveground parts of this species^20–22,48^. MWT could have influenced such diversity in the composition of root EO. The main component of all EOs from hogweed roots was p-cymene, a monoterpene hydrocarbon with strong biological properties, which is the main component of many known and used EOs^49^, including sunflower^50^. The biologically active compounds found in Sosnowski hogweed EO (which also have allelopathic properties) also change sugar metabolism, which has been previously observed^51^. Another main ingredient of oils with strong biological properties is myristicin. Interestingly, myristicin is a psychoactive EO ingredient from, for example, nutmeg and has anti-inflammatory, bactericidal, and anticancer potential^52^.

In the tested root EOs, none of the strongly photosensitizing compounds present in the aerial parts of hogweed, e.g., psoralen, bergapten, isobergapten, isopimpinellin, sphondin, and xanthotoxin^14,15^ was identified. The reason may be that the molecules of these compounds have such a high polarity and molecular weight that they are difficult to volatile with water vapor and do not occur in EOs. However, the root EOs contained alantolactone, an ingredient with strong pharmacological properties. It has antibacterial, anti-inflammatory, and antiviral properties. Its positive effect has even been proven in the treatment of colon cancer^53^. Interestingly, aliphatic alcohols with an irritating effect in hogweed root EO constituted a much smaller share than in seed EO, where they constitute one of the two main groups of volatile compounds^48^.

No analogy was observed in the composition of OE from hogweed roots in terms of dominant components, depending on the MWT time. However, the composition of EOs indicates their potential biological properties, which would be advisable to investigate in the future. The biological potential is influenced not only by ingredients such as furanocoumarins, aliphatic and unsaturated alcohols but also by aliphatic esters and aldehydes and ketones, including carvone, thymol and carvacrol methyl ether, piperitone, eugenol, and its derivatives methyleugenol and isoeugenol.

On the other hand, regarding the composition of sugar and fatty acids in the hogweed roots, we observed MWT-caused changes. As proved by the thermogram analyses, MWT increased the temperature of S. hogweed tissues and caused high-temperature stress, as confirmed by Kratchanova et al.^54^. Our study recorded high glucose and fructose concentrations for samples MWT for 15 min. In this case, MWT could lead to the hydrolision of cellulose, which is one of the main components of the cell wall or/also the hydrolision of starch, the major carbohydrate storage in plants. Starch content is seriously affected by adverse environmental stresses, including drought, salinity, osmotic stress, and low and high temperatures^55^. Hydrolysis of starch to oligosaccharides and that of oligosaccharides to monosaccharides, mainly reducing sugars, resulting from high temperature, may be responsible for increased concentration of sugars in plant tissue^56^. High concentrations of saccharose and maltose after 15 min of MWT indicate that they may be intermediate products resulting from polysaccharides, which are then broken down into monosaccharides.

Additionally, sucrose accumulation improves the water level in cells, protects cell membrane functions, and stabilizes subcellular structures^57^. Increased sucrose content in lettuce protects photosynthetic cells' structural and functional integrity^58^. The results obtained for the composition of fatty acids (FA) in the root tissue of *S. hogweed* following MWT indicate that the greatest increase was in the content of FA 16:0 and a significant decrease in the content of FA 18:2. Analogous observations were made for the desert shrub *Atriplex lentiformis*, in which high temperature caused an increase in the amount of saturated FA and a decrease in the amount of unsaturated FA^59^. Additionally, higher content of C16:0 and C18:0 may increase membrane rigidity^60^.

Changes in the degree of the saturation of the polyunsaturated fatty acids content can regulate the membrane's fluidity under heat stress^61^. In our research, the increase in the ratio of FA 18:3 to 18:2 was demonstrated after MWT. The highest values were observed for 5–10 min treatments. The lowest U/S ratio values were recorded 5 and 7.5 min after MWT. These results indicate that more saturated fatty acids are produced while, at the same time, the content of unsaturated fatty acids decreases. Our results are consistent with those obtained for rice exposed to high temperatures, which reduced the U/S ratio by 44%^62^.

In conclusion, the microwave-treated (MWT) Sosnowski hogweed plants, regardless of the treatment time, do not regrow up to fourteen days after treatment. The MWT causes an increase in the tissue temperature from 35 °C at 2.5 min MWT treatment up to 85 °C at 15 min MWT treatment, which reduces the dry matter of roots and causes heat stress. As an effect, root tissues undergo biochemical degradation. High glucose and fructose concentrations are recorded for the 15 min MWT samples. Also, following the MWT, more saturated fatty acids are produced while the content of unsaturated fatty acids decreases. In general, the exposure time of MWT of hogweed rhizomes causes changes in essential oil composition in favor of a greater composition diversity and a higher content of compounds with stronger properties than mono- or sesquiterpenes. The MWT does not affect soil ecotoxicity, as proved by the three biotests, which is a vital environmental benefit of our method. Our results could be a starting point for further studies on the effect of MWT on S. hogweed plants of different ages and located in different habitats. Moreover, further studies on the effect of microwaves on S. hogweed root essential oil carried out on other hogweed populations and in controlled conditions seem interesting, especially for analyzing aldehydes and ketones, the compounds of oil of biological activity.

### Supplementary Information

Supplementary Table 1.

## Data Availability

The datasets used and analyzed during the current study are available from the corresponding author (Krzysztof Słowiński) on reasonable request. Voucher specimens of Heracleum sosnowskyi Manden. (Voucher No. HERSO-07-2022) are deposited in a herbarium of the Department of Agroecology and Plant Production with access to deposited material. Dr. Beata Grygierzec identified specimens. Permission was obtained from the Municipal Greenery Board in Kraków to collect plant samples.
